# The prognostic values of m6A RNA methylation regulators in uveal melanoma

**DOI:** 10.1186/s12885-020-07159-8

**Published:** 2020-07-18

**Authors:** Jing Tang, Qi Wan, Jianqun Lu

**Affiliations:** 1 Department of Ophthalmology, The People’s Hospital of Leshan, city, Leshan, China; 2 State Key Laboratory of Ophthalmology, Zhongshan Ophthalmic Center, Sun Yat-Sen University, Guangzhou, 510064 China

**Keywords:** Uveal melanoma, m6A regulators, biomarker, Survival analysis

## Abstract

**Background:**

The aim of this study was to identify gene signatures and prognostic values of m6A methylation regulators in uveal melanoma (UM).

**Methods:**

The RNA sequencing dataset and corresponding clinical information were downloaded from TCGA and GEO database. Based on the expression of m6A RNA methylation regulators, the patients were further clustered into different groups by applying the “ClassDiscovery” algorithm. Best survival analysis was performed to select prognostic m6A regulators and multivariate cox regression analysis was applied to constructed m6A regulators signature. The association between mutations and m6A regulators was assessed by Kruskal−Wallis tests and clinical characteristics were examined by using chi-square test.

**Results:**

Totally, we identified two molecular subtypes of UM (C1/2) by applying consensus clustering to m6A RNA methylation regulators. In contrast to the C1 subtype, the C2 subtype associates with a better prognosis, has higher percentage of subtype 1 and lower percentage of Monosomy 3 which have been regarded as the well established prognostic markers for UM. The malignant hallmarks of mTORC1 signaling, oxidative phosphorylation, interferon-a response and apoptosis signaling are also significantly enriched in the C1 subtype. Moreover, a 3-m6A regulators signature was constructed by multivariate cox regression analysis method, which closely correlated with chromosome 3 status, subtype 1 of UM and can robustly predict patients’ overall survival time.

**Conclusions:**

m6A RNA methylation regulators take a crucial role in the potential malignant progression and prognostic value of UM and might be regarded as a new promising biomarker for UM prognosis and treatment strategy development.

## Background

Uveal melanoma is the most common type of malignant tumor of the adult eye, with an overall mortality rate of 50% [1, 2]. The prognosis for patients of UM remains poor, though advances in diagnosis and treatment have been reported [3, 4]. Therefore, it is important to explore the molecular mechanism underlying the survival events of UM and identify new prognostic factors and therapeutic targets.

It is well known that both DNA and histone proteins control gene expression by dynamic and reversible chemical modifications. RNA modification, like DNA and protein modification, is dynamically regulated by methyl-transferases [5]. The most prevalent RNA methylation is N6-methyladenosine (m6A), which exists in about 25% of transcripts at the genome-wide level and was firstly discovered in the 1970s. m6A RNA methylation regulators modify translocation, stability, RNA splicing and translation [6]. m6A is dynamically regulated by the ‘writers’ (RNA methyltransferases), such as METTL14, METTL3 and WTAP, is removed by ‘erasers’ (the demethylases), such as ALKBH5 and FTO, and ‘readers’ (the binding proteins), such as YTHDF2 and YTHDF1 [7]. RNA methyltransferases, the demethylases, and the binding proteins are often upregulated in a variety of human cancer types, increasing the expression of Oncogenes and Oncoproteins, augmenting the proliferation, progression, and metastasis of cancer cells [8].

m6A modification not only plays a vital role in the pathogenesis of a variety of human disease including obesity, neuronal disorders and immunological disease, but also has been shown to contribute to tumor initiation and promote progression of cancer and recurrence [9]. In addition, growing evidence suggests that gene mutation and abnormal expression of m6A regulators are intimately associated with the malignant progression of various cancers [10]. Although it is recognized that RNA methylation plays a critical role in different types of cancers, little is known about the relationship between m6A-related genes and UM.

Hence, in this study, we systematically evaluated the expression of m6A regulators in 80 UM samples from The Cancer Genome Atlas (TCGA) dataset as well as the association between the genetic alterations and clinical characteristics and validation in 28 UM samples from Gene Expression Omnibus (GEO) dataset. We found that the expression of m6A regulators plays critical roles in the malignant process of UM, and we identified three m6A regulators as potential biomarkers through their prognostic signatures.

## Methods

### Data processing

The RNA sequencing and mutation expression dataset as well as the corresponding clinical information of 80 uveal melanoma patients were downloaded from TCGA (http://cancergenome.nih.gov/). GSE84976 dataset consist of 28 uveal melanoma patients obtained from GEO (https://www.ncbi.nlm.nih.gov/geo), which was used for validation dataset. Firstly, the probe IDs were be transformed into official gene symbols based on the platform. When multiple probe IDs were matched to the same gene symbol, the probe ID with max expression value was selected to represent that gene. Then, the raw matrix data were normalized by log2(x + 1) conversion.

### Consensus clustering of m6A regulators

We first selected thirteen m6A RNA methylation regulators from previously published articles [7, 9, 10], and then we restricted the TCGA and GEO downloaded datasets to those features. Based on the expression of m6A regulators, we clustered the uveal melanoma patients into different subgroups. Principal component analysis was performed to determine whether these m6A regulators could definitely divide the samples into different uveal melanoma subgroups. To evaluate the interactive relationships among m6A regulators, correlation analyses of m6A regulators was applied and we also mapped the m6A regulators to the STRING database (http://stringdb.org). To investigate the pathways enriched in the different subgroups. we performed biology process (BP) and cancer hallmark pathway enrichment analysis. Firstly, patients of UM were classified into different subgroups and differentially analyses were calculated. Then, an ordered list of all genes was generated by the their log2 fold change value. GSEA was performed to assesses the functions associated with different subtypes.

### Prognostic signature building and risk survival analysis

The association between the m6A regulators and overall survival (OS) of melanoma patients was analyzed. Best survival analysis was performed to select the prognostic m6A regulators. Then, multivariate cox regression analysis method was used to construct prognostic signature with the selected m6A regulators. For the risk formula and the risk score is generated as follows: Risk score = ${\sum}_{i=1}^{\mathrm{N}}\left({coef}_i\times {expr}_i\right)$. Based on the risk model, the risk score of each sample was calculated. The patients were divided into high-risk group and low-risk group by using the median cutoff of risk score. The survival curves of Kaplan-Meier were drawn and the differences among groups were compared by log-rank tests. The area under the curve (AUC) of ROC curve was used to evaluate the 5-year overall survival predictive accuracy of the model.

### Statistical analysis

All statistical analyses were conducted using R (v.3.5.2). The samples in UM were clustered by applying the “ClassDiscovery” package. GSEA analysis was performed by using “clusterProfiler” package. The statistical significance of risk score distribution including chromosome 3 status, subtype, SF3B1 status, BAP1 status and immune infiltration was estimated by Kruskal−Wallis tests. Differences in the expression of m6A regulators between mutant and wildtype of top 5 mutated genes were performed by multiple testing and the corrected *p*-value was calculated with the Benjamini-Hockberg method. The correlation coefficient of expression of m6A regulators was calculated by Spearman method. The associated between m6A regulatory genes and clinicopathological characteristics were analyzed with Fisher exact test or Pearson Chi-square test appropriately. Univariate and multivariate Cox regression analyses were performed to determine the prognostic value of the risk score and various clinical characteristics. The Kaplan–Meier survival analysis was applied to compare the overall survival of the patients in the different groups or in the low- and high-risk groups. The hazard ratios (HR) and 95% confidence intervals (95% CI) of the prognostic factors were calculated. *P* < 0.05 was regarded as statistically significant in all statistical tests.

## Results

### Subgroup analysis of m6A regulators

**Fig. 1:**
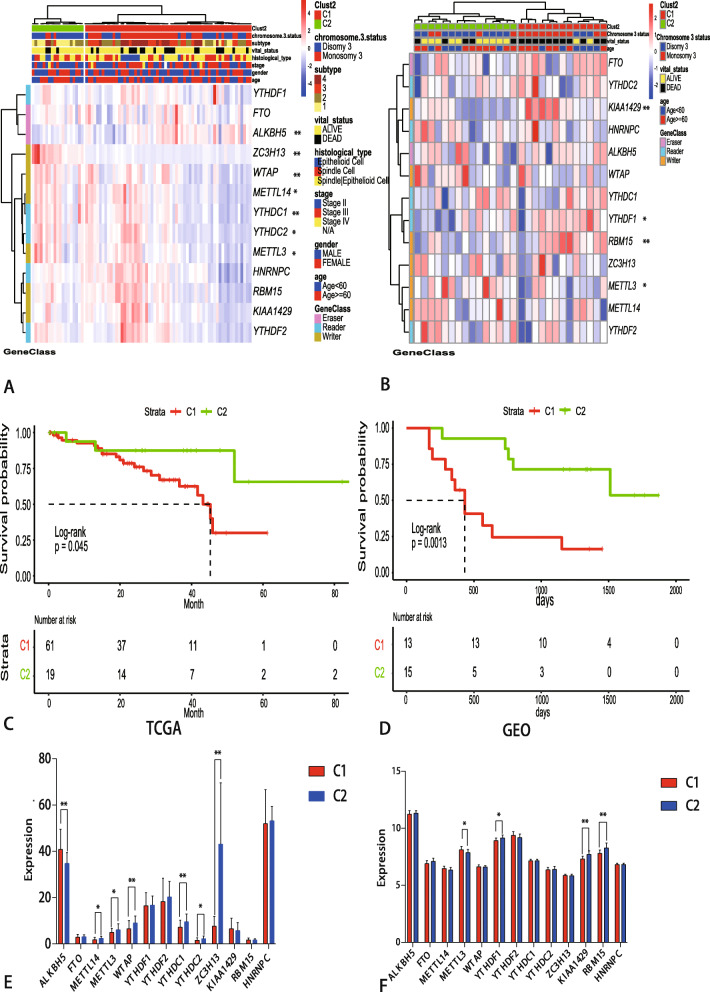
Expression of m6A RNA methylation regulators in uveal melanoma from the different database. **a**-**b** Heatmap and clinicopathologic features of the two clusters (C1/2) defined by the m6A RNA methylation regulators consensus expression downloaded from TCGA and GEO database. **c**-**d** Differential overall survival of uveal melanoma in the C1/2 subtypes (**e**-**f**) The mean expression levels of m6A RNA methylation regulators across the sample belonging to the C1/C2 group. * *P* < 0.05, ** *P* < 0.01

As a result, the expression of thirteen m6A RNA methylation regulators and clinicopathological characteristics associated to UM patients were obtained from TCGA and GEO. Based on “ClassDiscovery” algorithm, 80 UM patients from TCGA and 28 UM patients from GEO can be identified two clusters of groups, respectively (Fig. 1a and b). Then, we contrasted the clinical features of these two subgroups, namely, C1 and C2. The subgroups analysis of clinical characteristics showed that Chromosome 3 status and subtype of UM have significant differences (Table 1). The others clinical characteristics like age, gender, TNM and stage have no statistical significance. To find out the potential correlation of overall survival with C1 and C2. Kaplan-Meier survival analysis was performed and the curves showed that overall survival of samples in C2 is longer than the samples in the C1 group (Fig. 1c, d). Then, expression levels of thirteen m6A RNA methylation regulators in UM patients with different C1/2 groups were shown in Fig. 1e, f.

**Table 1:** Clinicopathological characteristics of C1/2 molecular subtypes

**TCGA**	**level**	**C1**	**C2**	**p**	**test**
n		61	19		
age (%)	Age < 60	27 (44.3)	9 (47.4)	1.000	Chisq Test
	Age > =60	34 (55.7)	10 (52.6)		
gender (%)	FEMALE	25 (41.0)	10 (52.6)	0.529	Chisq Test
	MALE	36 (59.0)	9 (47.4)		
M (%)	m0	39 (66.1)	12 (63.2)	0.972	Chisq Test
	m1	3 (5.1)	1 (5.3)		
	mx	17 (28.8)	6 (31.6)		
N (%)	n0	40 (65.6)	12 (66.7)	1.000	Chisq Test
	nx	21 (34.4)	6 (33.3)		
T (%)	t2	9 (14.8)	5 (26.3)	0.225	Chisq Test
	t3	23 (37.7)	9 (47.4)		
	t4	29 (47.5)	5 (26.3)		
stage (%)	N/A	1 (1.6)	0 (0.0)	0.238	Chisq Test
	Stage II	26 (42.6)	13 (68.4)		
	Stage III	31 (50.8)	5 (26.3)		
	Stage IV	3 (4.9)	1 (5.3)		
histological_type (%)	Epithelioid Cell	10 (16.4)	3 (15.8)	0.888	Chisq Test
	Spindle Cell	22 (36.1)	8 (42.1)		
	Spindle Cell|Epithelioid Cell	29 (47.5)	8 (42.1)		
vital_status (%)	ALIVE	41 (67.2)	16 (84.2)	0.255	Chisq Test
	DEAD	20 (32.8)	3 (15.8)		
subtype (%)	subtype1	5 (8.2)	10 (52.6)	0.000	Chisq Test
	subtype2	17 (27.9)	6 (31.6)		
	subtype3	21 (34.4)	1 (5.3)		
	subtype4	18 (29.5)	2 (10.5)		
chromosome.3.status (%)	Disomy 3	22 (36.1)	16 (84.2)	0.001	Chisq Test
	Monosomy 3	39 (63.9)	3 (15.8)		
**GEO**	**level**	**C1**	**C2**	**p**	**test**
n		15	13		
Age (%)	Age < 60	8 (53.3)	4 (30.8)	0.412	Fisher exact test
	Age > =60	7 (46.7)	9 (69.2)		
vital_status (%)	ALIVE	10 (66.7)	2 (15.4)	0.019	Fisher exact test
	DEAD	5 (33.3)	11 (84.6)		
Chromosome.3.status (%)	Disomy 3	11 (73.3)	3 (23.1)	0.023	Fisher exact test
	Monosomy 3	4 (26.7)	10 (76.9)		
Metastasis (%)	No	11 (73.3)	4 (30.8)	0.061	Fisher exact test
	Yes	4 (26.7)	9 (69.2)		

*M* Metastasis, *N* Lymph Node, *T* Tumor size

### Gene mutation and m6A regulators

**Fig. 2:**
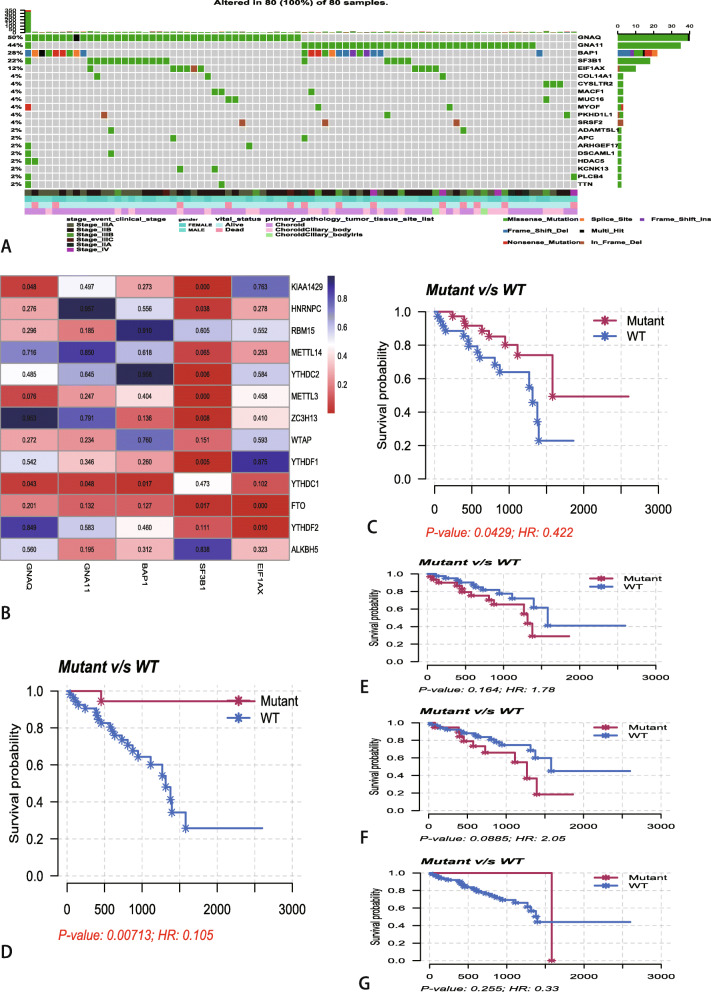
Relationships between mutated genes and mRNA expression levels of thirteen m6A regulators. **a** The waterfall plots of top 20 mutated genes in 80 UM samples at TCGA database. **b** The subgroup analysis the subgroup analysis of m6A regulators between mutant and wildtype of top 5 mutated genes. The blue and the red colors in heatmap represent higher and lower corrected *p*-value, respectively. **c**-**g** Kaplan-Meier survival analysis for GNAQ (**c**), SF3B1(**d**), GNA11(**e**), BAP1 (**f**) and EIF1AX (**g**) mutated genes

Then, we assessed the relationship between gene mutation and m6A regulators. we firstly identified the top 5 mutated genes based on the number of samples in which the genes are mutated in TCGA database, which was calculated by ‘maftools’ R package (Fig. 2a). The heatmap of differences in the expression of m6A regulators between mutant and wildtype of top 5 mutated genes indicated that SF3B1 was the most significantly regulated the expression of m6A regulators (Fig. 2b). The heatmap showed that the expression levels of ALKBH5, FTO, WTAP, YTHDF1, YTHDF2, YTHDC2 and KIAA1429 are significant differences between mutant-SF3B1 and wildtype-SF3B1. Kaplan-Meier analysis of these 5 mutant genes showed that only GNAQ (Fig. 2c) and SF3B1 (Fig. 2d) have significant differences with overall survival. As for GNA11 (Fig. 2e), BAP1 (Fig. 2f) and EIF1AX (Fig. 2g), the Kaplan-Meier analysis showed that there is on significantly differences between mutant and wildtype.

### Clustered molecular subtype of uveal melanoma

**Fig. 3:**
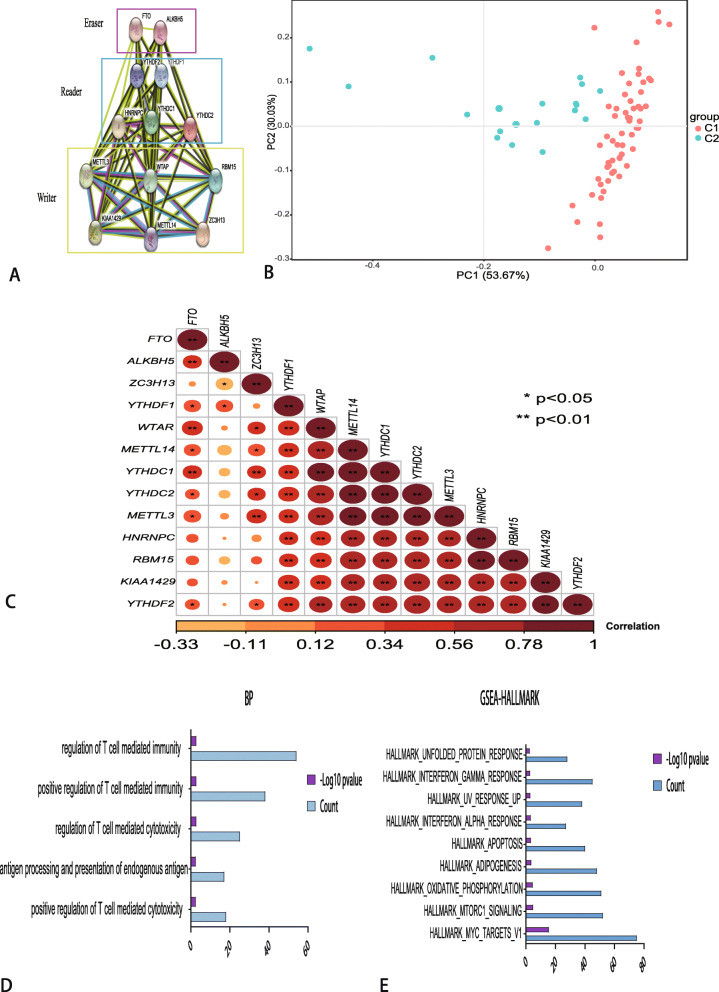
Interaction among m6A RNA methylation regulators and functional annotation of uveal melanoma in C1/2 subtypes. **a** The m6A modification-related interactions among the 13 m6A RNA methylation regulators. **b** Principal component analysis of the total RNA expression profile in the TCGA dataset. **c** Spearman correlation analysis of the 13 m6A modification regulators. * *P* < 0.05, ** *P* < 0.01. **d** The top 5 biology process (BP) terms were actively enriched in C1 expression phenotype. X-axis means the number of genes enriched in pathways, Y-axis means BP pathways. **f** Cancer hallmark pathways revealed that 9 malignant hallmark pathways were significantly associated with the C1 subgroup expression phenotype. X-axis means the number of genes enriched in pathways, Y-axis means hallmark pathways

The above results revealed that the clustered molecular subtype was intimately related to the prognosis of uveal melanoma. For better understanding of the interrelations among the thirteen m6A regulators, we also analyzed the interrelation (Fig. 3a) and correlation (Fig. 3c) among these regulators. ALKBH5 seems to be the hub gene of the ‘Eraser’, and correlated or co-expressed with METTL3, WTAP, YTHDF2, M ETTL14, YTHDF1, YTHDC1, YTHDC2, RBM15, KIAA1429. The correlation analysis showed that these regulators were significantly positively correlated with each other. Principal components analysis showed that C1 samples and C2 samples in TCGA datasets could be well differentiated based on the expression of m6A regulators (Fig. 3b). To investigate biologic pathways shared by the different C1/2 subtype, we performed GSEA analysis. According to the following criteria: *p* value< 0.05 and normalized enrichment score: | NES | ≥1. 49 BP terms were differentially enriched in C1 expression phenotype. The top 5 BP terms indicated that pathways are commonly enriched T cell mediated pathways, including positive regulation of T cell mediated cytotoxicity, antigen processing and presentation of endogenous antigen, regulation of T cell mediated cytotoxicity, positive regulation of T cell mediated immunity and regulation of T cell mediated immunity (Fig. 3d). Moreover, the GSEA analysis of cancer malignant hallmarks of tumors showed that 9 terms including mTORC1 signaling, oxidative phosphorylation, interferon-a response and apoptosis signaling were significantly associated with the C1 subgroup expression phenotype (Fig. 3e).

### Identification and confirmation of m6A regulators signature

**Fig. 4:**
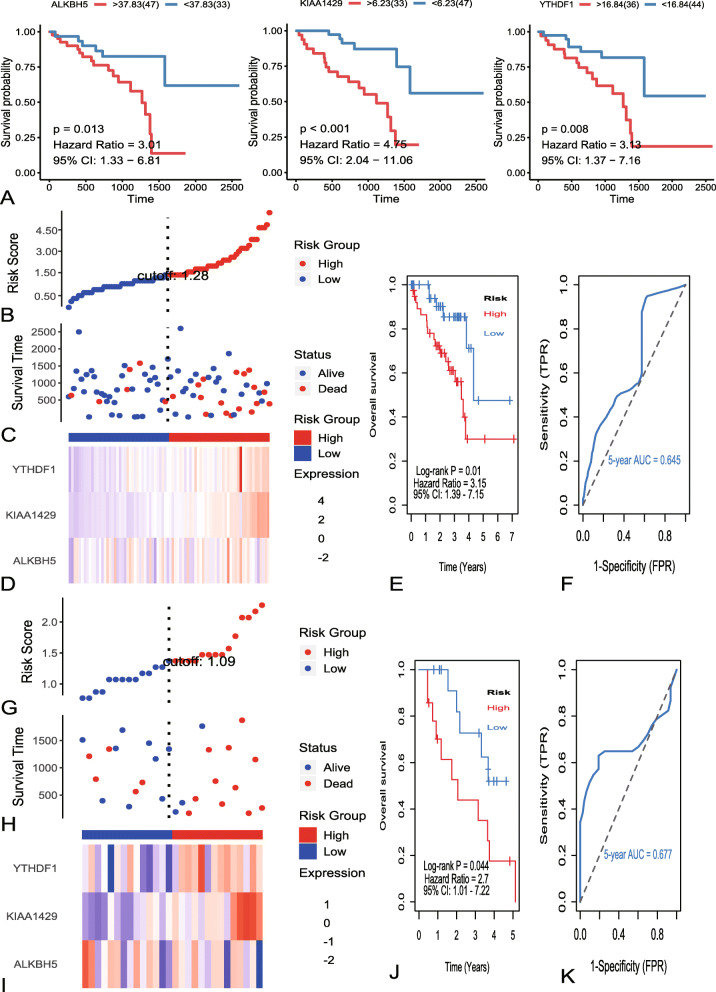
Identification and validation of m6A regulators signature. **a** Kaplan-Meier survival curves of ALKBH5, YTHDF1 and KIAA1429. **b** The distribution of risk score in TCGA dataset. The risk scores are arranged in ascending order from left to right. **c** The overall survival (OS) and vital status of patients. **d** The expression patterns of three identified m6A regulators for 80 patients in TCGA. **e** Kaplan–Meier survival curves of patients in the high-risk and low-risk groups. **f** The 5 years of the receiver operating characteristic (ROC) curve in TCGA dataset. **g** The distribution of risk score in GEO dataset. The risk scores are arranged in ascending order from left to right. **h** The overall survival (OS) and vital status of patients. **i** The expression patterns of three identified m6A regulators for 28 patients in GEO. **j** Kaplan–Meier survival curves of patients in the high-risk and low-risk groups. **k** The 5 years of the receiver operating characteristic (ROC) curve in GEO dataset

For better predict the clinical and pathologic outcomes of UM with m6A regulators. Firstly, best survival analysis was used to evaluate associations between m6A regulators and OS in TCGA dataset. Totally, three m6A regulators were seeded out (Fig. 4a). Then, we used the three m6A regulators to constructed risk system by multivariate cox regression analysis. The risk system reckons a risk score for each patient. The distributions of the risk scores, OS, vital status, and expression levels of corresponding 3 m6A regulators in TCGA dataset were shown in Fig. 4b-d. Next, UM samples were divided into a high-risk group (*n* = 40) and a low-risk group (*n* = 40) by applying the median value of the risk scores. Kaplan-Meier curves revealed that low risk group have a significant longer survival time than high risk (Fig. 4e). The ROC curve showed that the 5 years of AUC was 0.645 (Fig. 4f). To verify the predictive ability of the three m6A regulators, validation analysis was performed in GEO dataset. The distributions of the risk scores, OS, vital status, and expression levels of corresponding 3 m6A regulators in GEO dataset were shown in Fig. 4g-i. The curve of Kaplan-Meier revealed that there is a significant difference between high-risk and low-risk group with log-rank test of *p* = 0.044 (Fig. 4j). The 5 years of AUC was 0.677 (Fig. 4k). The subgroups analysis of clinical characteristics between low- and high- risk groups showed that chromosome 3 status, subtype, and vital status in TCGA and GEO have significant differences (Table 2).

**Table 2:** The subgroups analysis of clinical characteristics between low- and high- risk groups

**TCGA**	**level**	**High_risk**	**Low_risk**	**p**	**test**
n		40	40		
age (%)	Age < 60	16 (40.0)	20 (50.0)	0.500	Chisq Test
	Age > =60	24 (60.0)	20 (50.0)		
gender (%)	FEMALE	14 (35.0)	21 (52.5)	0.176	Chisq Test
	MALE	26 (65.0)	19 (47.5)		
M (%)	m0	25 (64.1)	26 (66.7)	0.588	Chisq Test
	m1	3 (7.7)	1 (2.6)		
	mx	11 (28.2)	12 (30.8)		
N (%)	n0	25 (64.1)	27 (67.5)	0.935	Chisq Test
	nx	14 (35.9)	13 (32.5)		
T (%)	t2	5 (12.5)	9 (22.5)	0.440	Chisq Test
	t3	18 (45.0)	14 (35.0)		
	t4	17 (42.5)	17 (42.5)		
stage (%)	N/A	1 (2.5)	0 (0.0)	0.481	Chisq Test
	Stage II	20 (50.0)	19 (47.5)		
	Stage III	16 (40.0)	20 (50.0)		
	Stage IV	3 (7.5)	1 (2.5)		
histological_type (%)	Epithelioid Cell	10 (25.0)	3 (7.5)	0.010	Chisq Test
	Spindle Cell	9 (22.5)	21 (52.5)		
	Spindle Cell|Epithelioid Cell	21 (52.5)	16 (40.0)		
vital_status (%)	ALIVE	23 (57.5)	34 (85.0)	0.014	Chisq Test
	DEAD	17 (42.5)	6 (15.0)		
subtype (%)	subtype1	3 (7.5)	12 (30.0)	0.000	Chisq Test
	subtype2	9 (22.5)	14 (35.0)		
	subtype3	10 (25.0)	12 (30.0)		
	subtype4	18 (45.0)	2 (5.0)		
chromosome.3.status (%)	Disomy 3	12 (30.0)	26 (65.0)	0.004	Chisq Test
	Monosomy 3	28 (70.0)	14 (35.0)		
**GEO**	**level**	**High_risk**	**Low_risk**	**p**	**test**
n		14	14		
Age (%)	Age < 60	5 (35.7)	7 (50.0)	0.703	Fisher exact test
	Age > =60	9 (64.3)	7 (50.0)		
vital_status (%)	ALIVE	3 (21.4)	9 (64.3)	0.052	Fisher exact test
	DEAD	11 (78.6)	5 (35.7)		
Chromosome.3.status (%)	Disomy 3	4 (28.6)	10 (71.4)	0.053	Fisher exact test
	Monosomy 3	10 (71.4)	4 (28.6)		
Metastasis (%)	No	5 (35.7)	10 (71.4)	0.130	Fisher exact test
	Yes	9 (64.3)	4 (28.6)		

*M* Metastasis, *N* Lymph Node, *T* Tumor size

### Associations between risk score and clinical variables

**Fig. 5:**
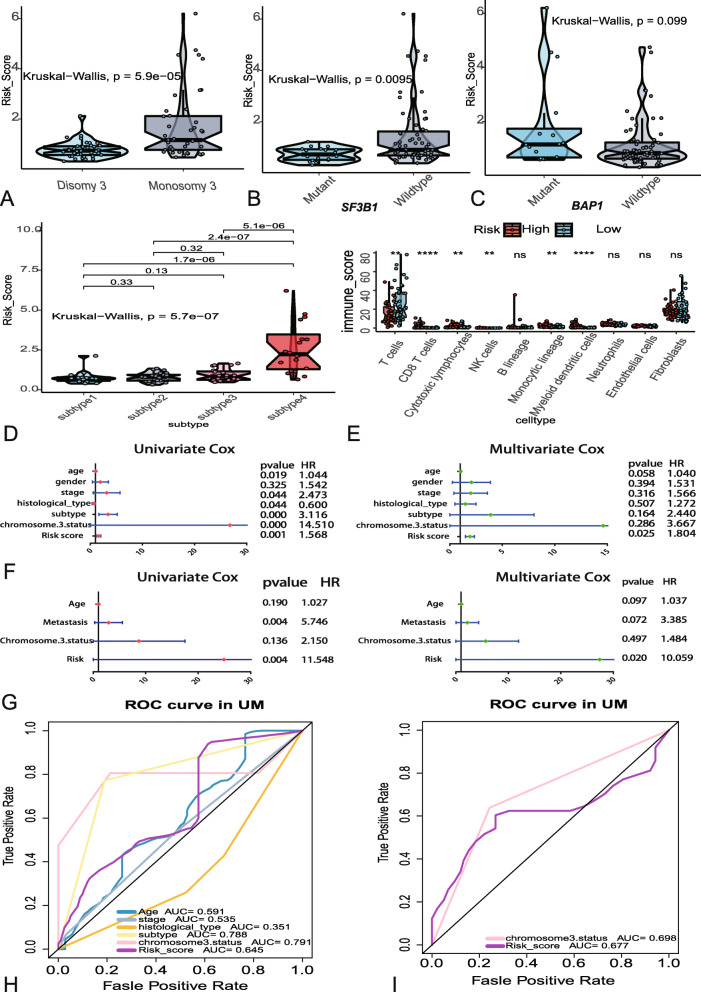
Associations between risk score and clinical variables. **a**-**d** The relationship between risk score distribution and clinical variables which contained chromosome 3 status (**a**), SF3B1mutated types (**b**), BAP1 mutated types (**c**) and subtype (**d**). **e** The different immune scores of 10 immune cells between the high and low risk UM patients. **f** Forest plots of risk score and clinical variables in TCGA dataset. **g** Forest plots of risk score and clinical variables in GEO dataset. **h** The 5 years area under the curve (AUC) of risk score and clinical variables associated with OS in TCGA. **i** The 5 years AUC of risk score and clinical variables associated with OS in GEO

The associations between risk score of m6A regulators and clinical variables such as chromosome 3 status, mutated SF3B1, mutated BAP1 and subtype were explored. The box plots showed that monosomy 3 have higher risk scores than disomy 3 (Fig. 5a), wildtype of SF3B1 own higher risk scores than mutant (Fig. 5b), and subtype 4 of UM have the highest scores than other subtypes (Fig. 5d). While, subgroup of mutated BAP1 manifested that there is on significant difference between mutant and wildtype (Fig. 5c). To evaluate the associations between risk score and immune microenvironment, “MCPcounter” package in R was applied to calculate the immune scores of immune cells. Subgroup analysis of immune cells showed that significant differences were founded in T cells, CD8 T cells, cytotoxic lymphocytes, natural killer (NK) cells, monocytic lineage and myeloid dendritic cells between high and low risk subgroups (Fig. 5e). Besides, univariate and multivariate logistic regression were used to compare the prognostic value of risk score and other clinical variables in TCGA and GEO datasets. The forest plots indicated that age, stage, histology, subtype, chromosome 3 status, metastasis and risk sore were significantly associated with OS in univariate analysis, but only the risk score were significantly correlated with OS in multivariate analysis (Fig. 5f-g). The 5 years AUC of age, stage, histology, subtype, chromosome 3 status and risk score in TCGA were 0.591, 0.535, 0.351, 0.788, 0.791 and 0.645 respectively (Fig. 5h). As for GEO, the 5 years AUC of chromosome 3 status and risk score were 0.698 and 0.677 (Fig. 5i).

## Discussion

The growing genome-wide studies demonstrated that most of the human genome is transcribed, which exists a complex network consist of large and small RNA molecules in human cells. However, only 1 to 2% of the transcripts have the capacity for protein translation [11–13]. In fact, post-transcriptional regulation at the RNA level through cis-and trans-mechanisms is essential to control the gene expression procedures that determine cellular function and cell fate [14]. To date, more than 150 chemical modifications have been described for RNA. Among them, m6A is the most prevalent posttranscriptional modification of eukaryotic mRNAs and long noncoding RNAs. Recent studies have indicated that m6A regulators have been shown to play important regulatory roles in diverse biological processes in human cancer [15]. However, despite of the increasing evidence for their implication in cancers, the potential role of m6A regulators in UM prognosis is little known about.

In this study, we demonstrated that the expression of m6A regulators is also intimately related to the prognosis and malignancy of UM. Based on the expression of m6A regulators, we identified two UM subgroups, namely C1/2 molecular subgroup, by applying consensus clustered method. The C1/2 molecular subgroup not only affected the clinical and prognosis features but also closely associated with biological signals and malignant hallmarks of UM. Survival analysis showed that C1 subgroup have worse overall survival than C2 subgroup. In addition, C1 subgroup have higher percentage of subtype 4 which have been proven the worst outcome subgroup in previous UM TCGA study [16]. GSEA analysis showed that positive regulation of T cell mediated pathways and malignant hallmarks such as mTORC1 signaling, oxidative phosphorylation, interferon-a response and apoptosis signaling positively activate in C1 subgroup. In fact, T cells like active CD 4 + and CD 8 + cells have antitumor immunity and therapy functions [17]. As to C1 molecular subtype, lots of malignant hallmark of pathways were enriched. Thus, it is reasonable to believe that clustered molecular subtypes C1/2 are closely correlated to the malignancy and prognosis of UM. Moreover, extensive researches also suggest that UM with monosomy 3 is associated with a dramatically poor prognosis, which consistent with our research [18–20]. The subgroups analysis of Chromosome.3.status showed that the percentage of Monosomy 3 in C1 molecular subgroup is much higher than this in C2 molecular subtype (Table 1). The different analysis of m6A regulators between C1/2 molecular subtype showed that “eraser” like ALKBH5, “writer” like METTL3, METTL14, and WTAP and “readers”, like YTHDF1 and YTHDF2 have significant differences. (Fig. 1e, f). The different expression of these m6A regulators may eventually lead to the various of survival outcomes [21]. Among the m6A regulators, previous studies indicated that the eraser ALKBH5 can induce breast cancer stem cell and glioblastoma stem-like cell proliferation and tumor initiation, [22] the writers METTL3 and METTL14 were reported to enhance glioblastoma growth and suppress Liver cancer metastasis, [23–25] the reader YTHDF1 and YTHDF2 induce cancer cell proliferation in colon cancer and lung oncogenic effects [26, 27]. These findings manifested that high or low expression of specific m6A regulators are related to misregulation of RNAs in tumors, and the same m6A regulator may take different functions in various tumors [28, 29].

By analyzing the mutation annotation files of the TCGA-UVM cohort, we identified 5 highly variant mutated genes and SF3B1 is the most significantly influence the expression of m6A regulators. SF3B1 (splicing factor 3subunit B1) mutations can be generally found in 10 to 21% of cases of UM. Previous researches have shown that SF3B1 mutations in UM patients are associated with favorable prognosis [30]. Survival analysis also indicated that SF3B1-mutated UM had a better survival than the SF3B1 wild-type. In our research, the results showed that the mutation of SF3B1 will generally significantly down-regulated the expression of m6A regulators, including “eraser” such as ALKBH5 and FTO; “writer” such as WTAP and KIAA1429; “reader” such as YTHDF1, YTHDF2 and YTHDC2 (Fig. 2b). Therefore, it easily envisaged that the mutant of SF3B1 may lead to down-regulate the expression of “eraser” such as ALKBH5 and FTO and finally result in a better survival in UM.

What’s more, we also distinguished a prognostic risk signature with three identified m6A regulators (ALKBH5, YTHDF1 and KIAA1429), which divided the overall survival of UM into high- and low-risk subgroups. Kaplan-Meier analyses indicated that high-risk subgroups with a poor survival. Stratified analysis of clinical characteristics between low- and high- risk groups also revealed that lots of risk factors like mortality rate, subtype 4 and monosomy 3 are take higher percentage in high-risk group (Table 2). Furthermore, UM patients in high risk group had higher immune infiltration than low risk group. The risk sores of monosomy 3, SF3B1-wildtype, and subtype 4 were respectively higher than disomy 3, SF3B1-mutated and subtype 1 in UM, which was consistent with previous researches. Notably, compared with the 5 years AUC values of previous prognostic markers (stage, subtype and chromosome 3 status), our signature can achieve similar accuracy value. Besides, only the risk score had significant associations with OS no matter in univariate or multivariate regression analysis. In sum, the signature we constructed might be regarded as a new promising biomarker which supply more simple and accurate clinical applications. For example, in human breast cancer cells, knockdown ALKBH5 contributed to significantly decrease the number of cancer stem cells and the opportunity of tumorigenesis. In addition, the high expression of ALKBH5 in glioblastoma can lead to stem-like cell proliferation and tumorigenesis [31].

## Conclusions

In summary, we firstly comprehensively evaluated the expression, potential function, and prognostic value of m6A regulatory genes in UM from TCGA dataset and have validated in GEO dataset, which should be helpful for UM early diagnosis and might be regarded as a new promising biomarker for UM prognosis and treatment.

## Data Availability

The datasets used and analysed during the current study available from the corresponding author on reasonable request.

## Abbreviations

TCGA: The Cancer Genome Atlas database
GEO: Gene Expression Omniniub
UM: Uveal melanoma
GO: Gene ontology
BP: Biology process
DEGs: Differently expressed genes
OS: Overall survival
AUC: The area under the curve
ROC: Receiver operating characteristic curves
